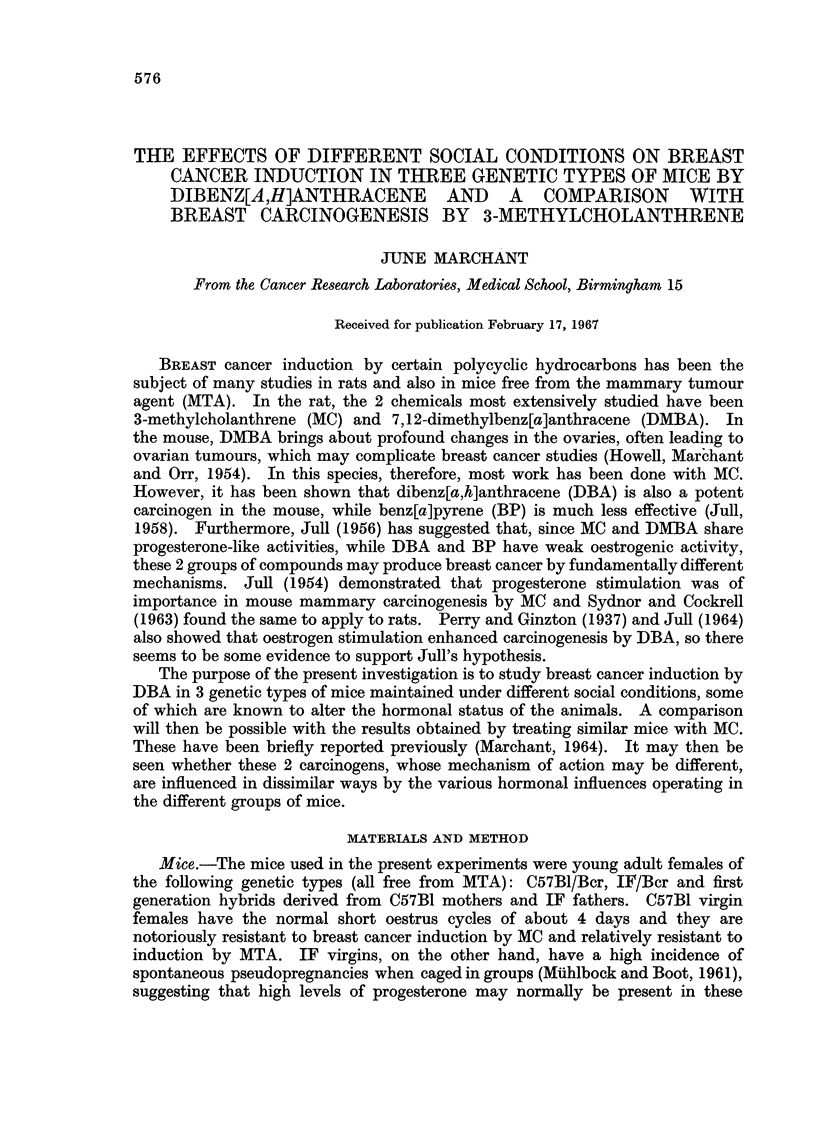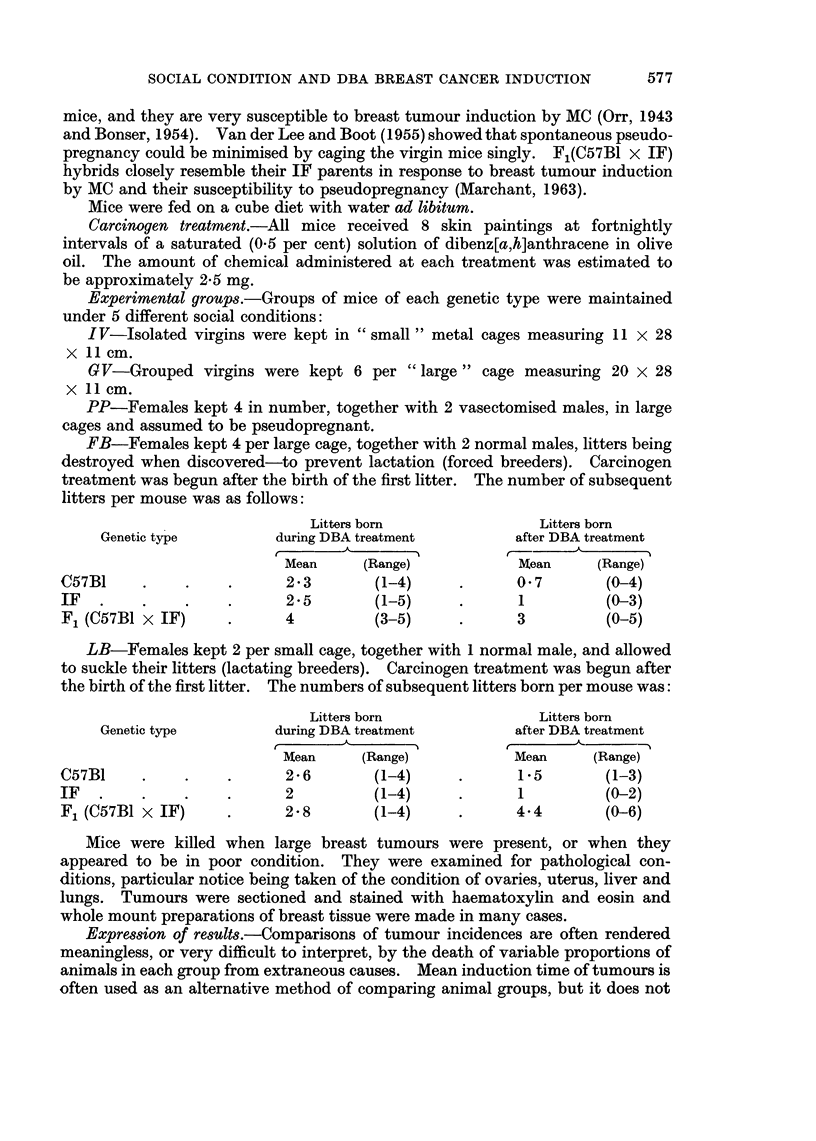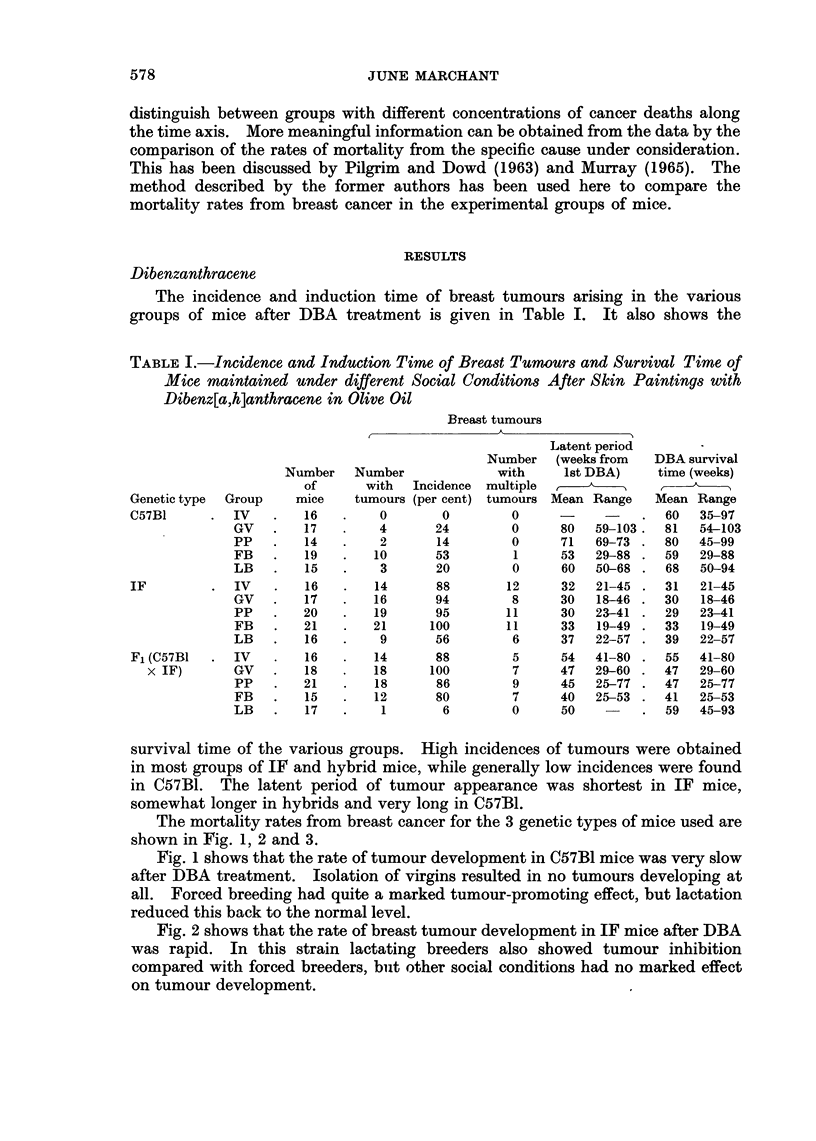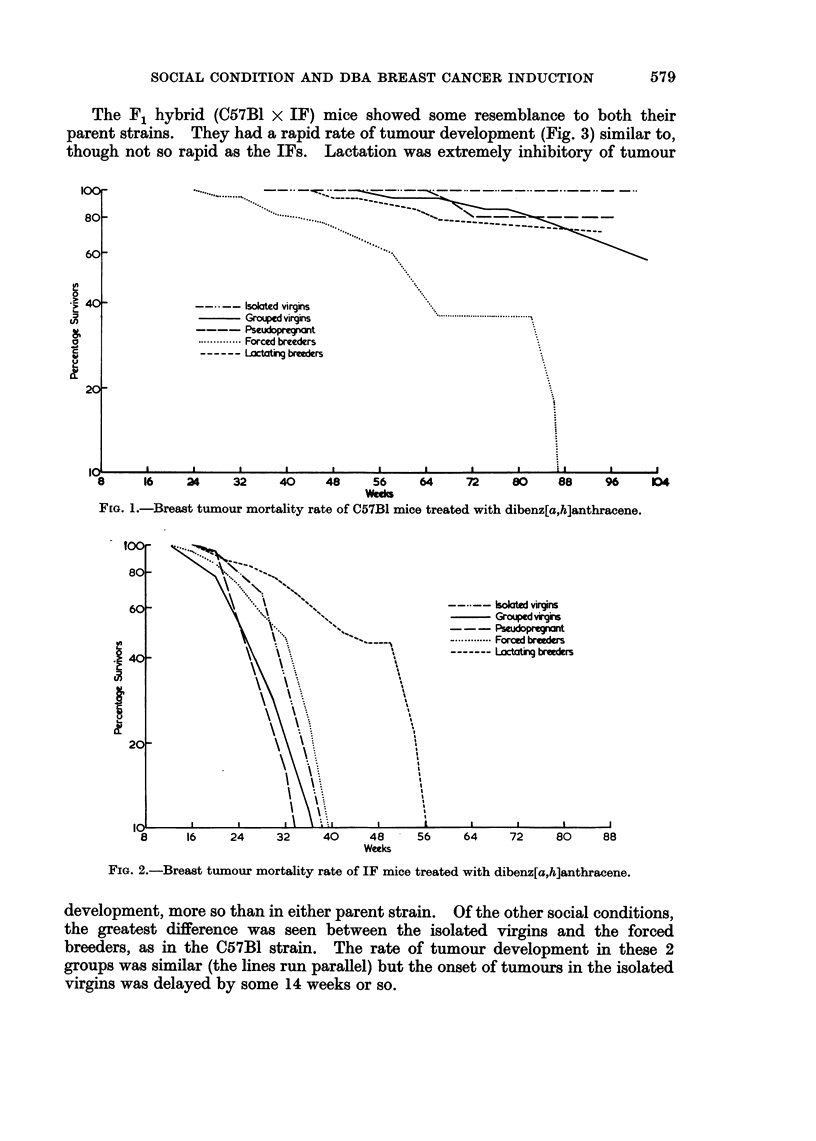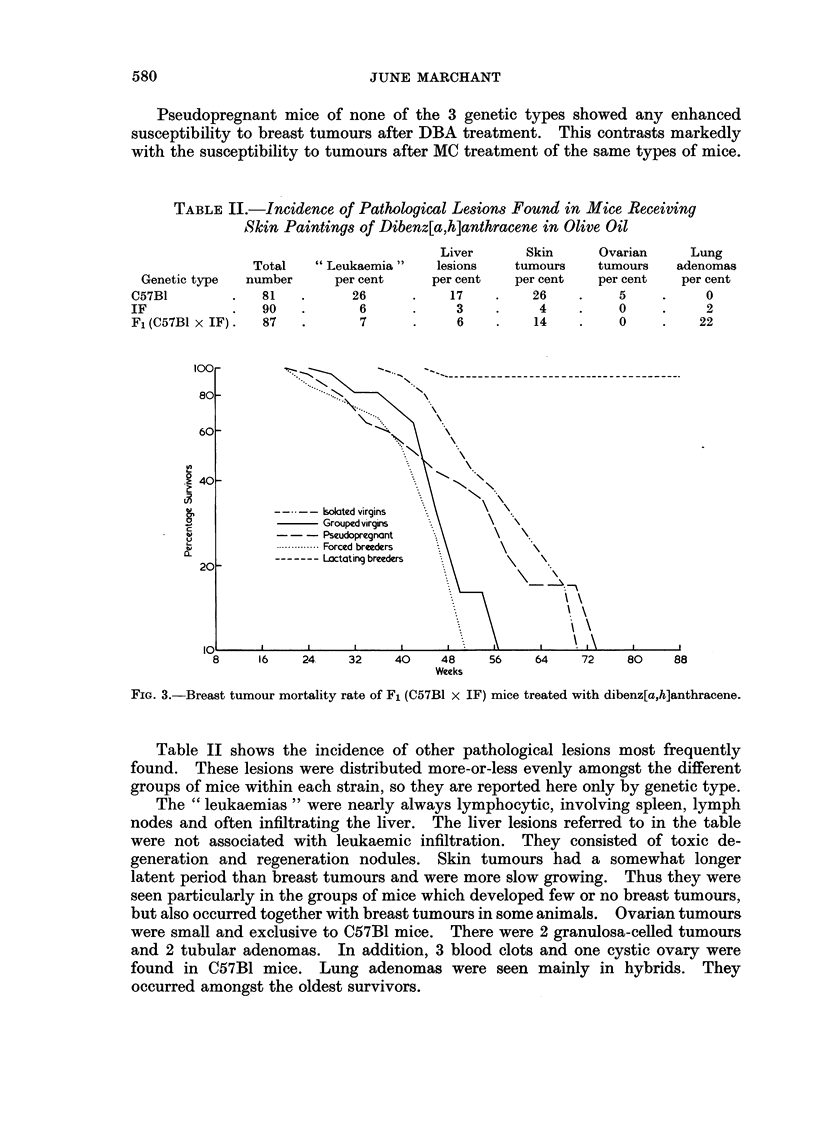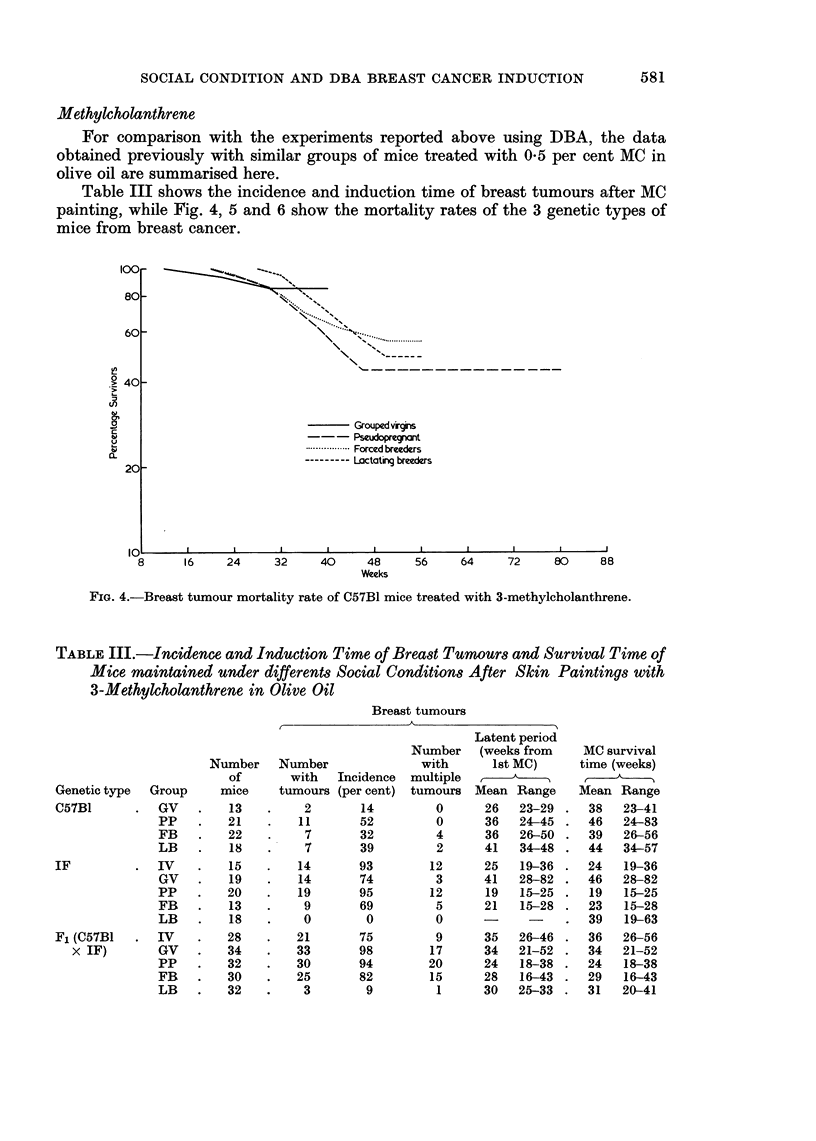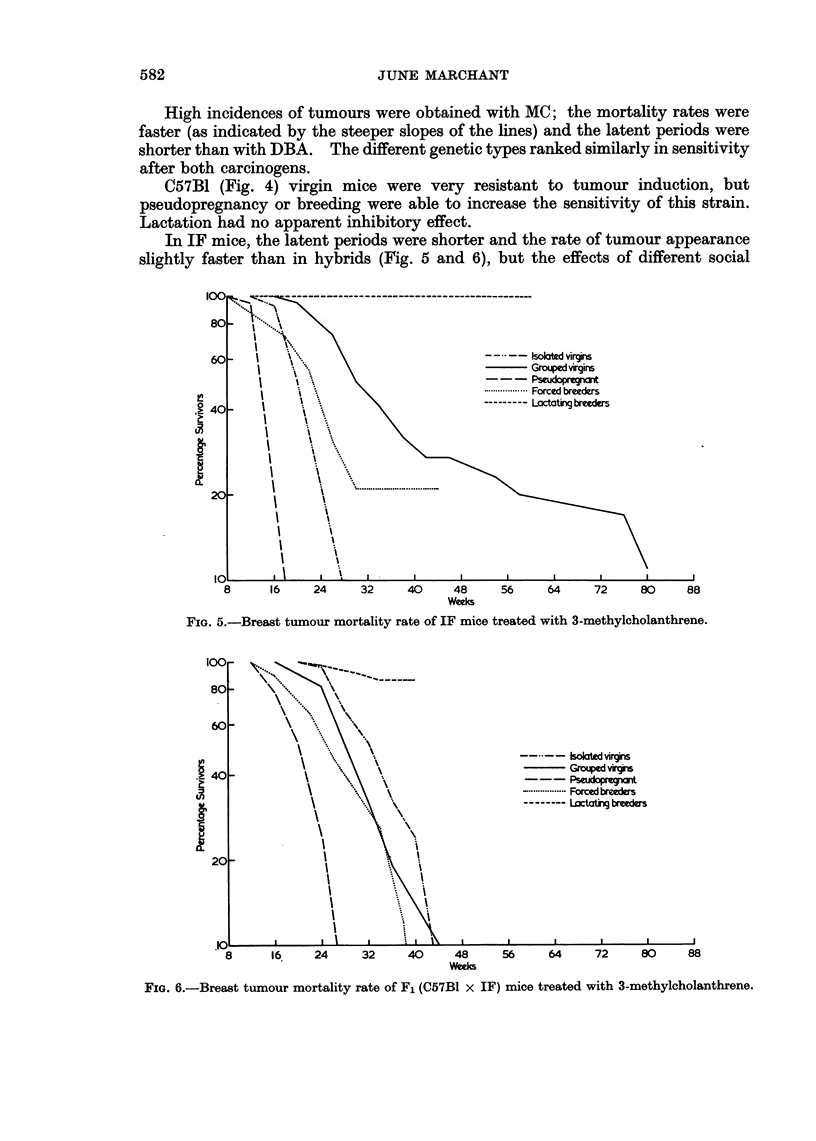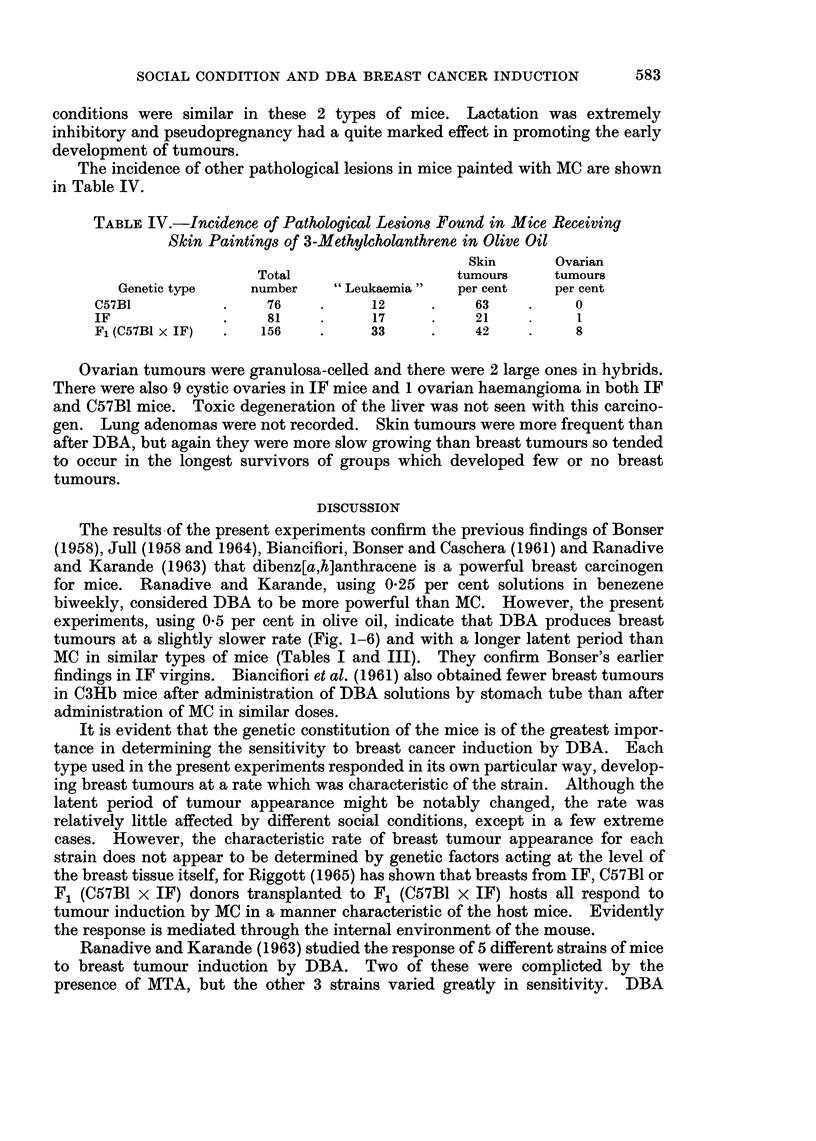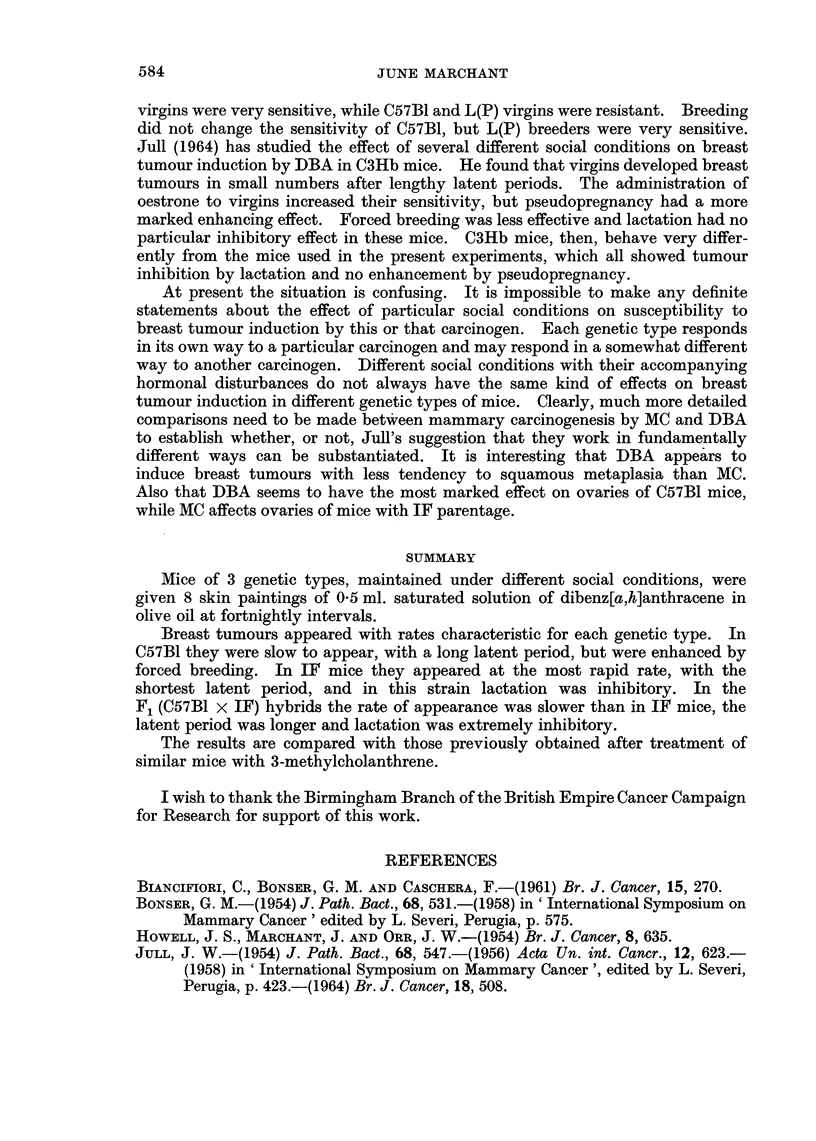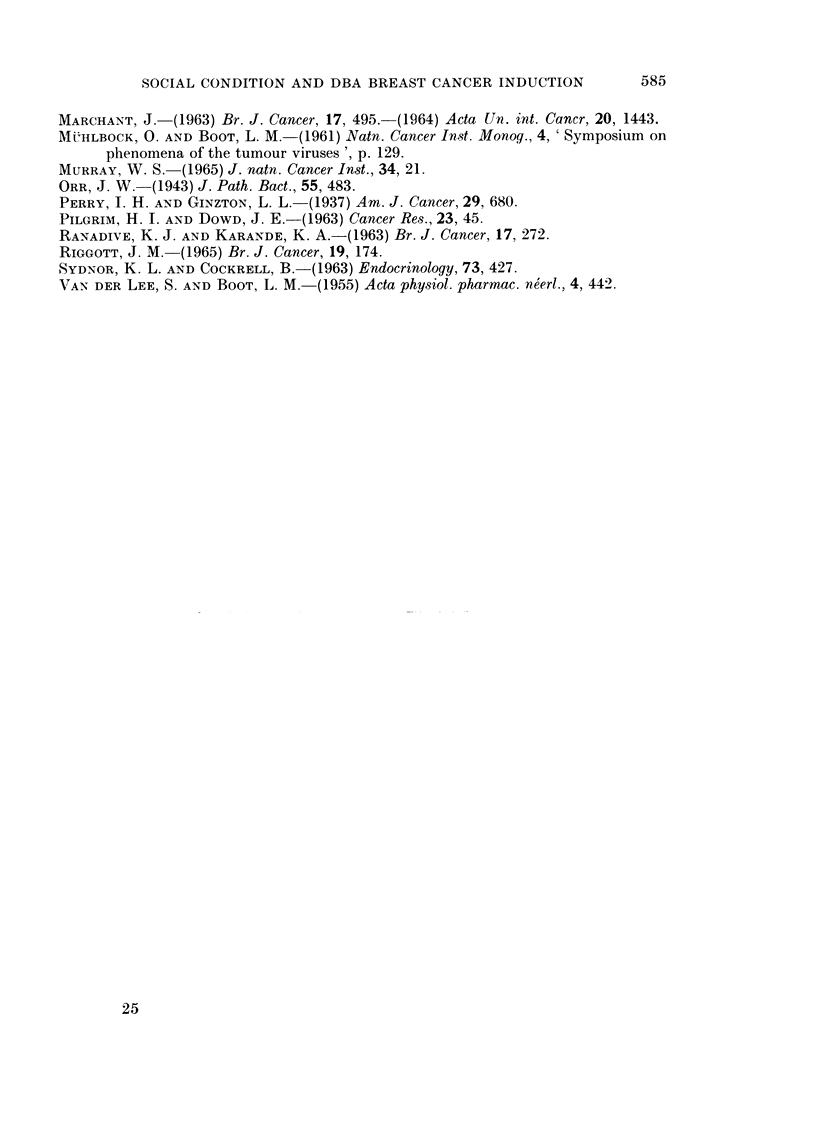# The effects of different social conditions on breast cancer induction in three genetic types of mice by dibenz[a,h]anthracene and a comparison with breast carcinogenesis by 3-methylcholanthrene.

**DOI:** 10.1038/bjc.1967.67

**Published:** 1967-09

**Authors:** J. Marchant


### THE EFFECTS OF DIFFERENT SOCIAL CONDITIONS ON BREAST CANCER INDUCTION IN THREE GENETIC TYPES OF MICE BY DIBENZ[*A*,*H*]ANTHRACENE AND A COMPARISON WITH BREAST CARCINOGENESIS BY 3-METHYLCHOLANTHRENE

JUNE MARCHANT

*From the Cancer Research Laboratories, Medical School, Birmingham* 15

Received for publication February 17, 1967

BREAST cancer induction by certain polycyclic hydrocarbons has been the subject of many studies in rats and also in mice free from the mammary tumour agent (MTA). In the rat, the 2 chemicals most extensively studied have been 3-methylcholanthrene (MC) and 7,12-dimethylbenz[*a*]anthracene (DMBA). In the mouse, DMBA brings about profound changes in the ovaries, often leading to ovarian tumours, which may complicate breast cancer studies (Howell, Marchant and Orr, 1954). In this species, therefore, most work has been done with MC. However, it has been shown that dibenz[*a*,*h*]anthracene (DBA) is also a potent carcinogen in the mouse, while benz[*a*]pyrene (BP) is much less effective (Jull, 1958). Furthermore, Jull (1956) has suggested that, since MC and DMBA share progesterone-like activities, while DBA and BP have weak oestrogenic activity, these 2 groups of compounds may produce breast cancer by fundamentally different mechanisms. Jull (1954) demonstrated that progesterone stimulation was of importance in mouse mammary carcinogenesis by MC and Sydnor and Cockrell (1963) found the same to apply to rats. Perry and Ginzton (1937) and Jull (1964) also showed that oestrogen stimulation enhanced carcinogenesis by DBA, so there seems to be some evidence to support Jull's hypothesis.

The purpose of the present investigation is to study breast cancer induction by DBA in 3 genetic types of mice maintained under different social conditions, some of which are known to alter the hormonal status of the animals. A comparison will then be possible with the results obtained by treating similar mice with MC. These have been briefly reported previously (Marchant, 1964). It may then be seen whether these 2 carcinogens, whose mechanism of action may be different, are influenced in dissimilar ways by the various hormonal influences operating in the different groups of mice.

### MATERIALS AND METHOD

*Mice*.—The mice used in the present experiments were young adult females of the following genetic types (all free from MTA): C57Bl/Bcr, IF/Bcr and first generation hybrids derived from C57Bl mothers and IF fathers. C57Bl virgin females have the normal short oestrus cycles of about 4 days and they are notoriously resistant to breast cancer induction by MC and relatively resistant to induction by MTA. IF virgins, on the other hand, have a high incidence of spontaneous pseudopregnancies when caged in groups (Mühlbock and Boot, 1961), suggesting that high levels of progesterone may normally be present in these mice, and they are very susceptible to breast tumour induction by MC (Orr, 1943 and Bonser, 1954). Van der Lee and Boot (1955) showed that spontaneous pseudopregnancy could be minimised by caging the virgin mice singly. $F_1$(C57Bl × IF) hybrids closely resemble their IF parents in response to breast tumour induction by MC and their susceptibility to pseudopregnancy (Marchant, 1963).

Mice were fed on a cube diet with water *ad libitum.*

*Carcinogen treatment.*—All mice received 8 skin paintings at fortnightly intervals of a saturated (0·5 per cent) solution of dibenz[*a,h*]anthracene in olive oil. The amount of chemical administered at each treatment was estimated to be approximately 2·5 mg.

*Experimental groups.*—Groups of mice of each genetic type were maintained under 5 different social conditions:

*IV*—Isolated virgins were kept in " small " metal cages measuring 11 × 28 × 11 cm.

*GV*—Grouped virgins were kept 6 per " large " cage measuring 20 × 28 × 11 cm.

*PP*—Females kept 4 in number, together with 2 vasectomised males, in large cages and assumed to be pseudopregnant.

*FB*—Females kept 4 per large cage, together with 2 normal males, litters being destroyed when discovered—to prevent lactation (forced breeders). Carcinogen treatment was begun after the birth of the first litter. The number of subsequent litters per mouse was as follows:

| Genetic type | Litters born during DBA treatment | | Litters born after DBA treatment | |
|---|---|---|---|---|
| | Mean | (Range) | Mean | (Range) |
| C57Bl | 2·3 | (1–4) | 0·7 | (0–4) |
| IF | 2·5 | (1–5) | 1 | (0–3) |
| $F_1$ (C57Bl × IF) | 4 | (3–5) | 3 | (0–5) |

*LB*—Females kept 2 per small cage, together with 1 normal male, and allowed to suckle their litters (lactating breeders). Carcinogen treatment was begun after the birth of the first litter. The numbers of subsequent litters born per mouse was:

| Genetic type | Litters born during DBA treatment | | Litters born after DBA treatment | |
|---|---|---|---|---|
| | Mean | (Range) | Mean | (Range) |
| C57Bl | 2·6 | (1–4) | 1·5 | (1–3) |
| IF | 2 | (1–4) | 1 | (0–2) |
| $F_1$ (C57Bl × IF) | 2·8 | (1–4) | 4·4 | (0–6) |

Mice were killed when large breast tumours were present, or when they appeared to be in poor condition. They were examined for pathological conditions, particular notice being taken of the condition of ovaries, uterus, liver and lungs. Tumours were sectioned and stained with haematoxylin and eosin and whole mount preparations of breast tissue were made in many cases.

*Expression of results.*—Comparisons of tumour incidences are often rendered meaningless, or very difficult to interpret, by the death of variable proportions of animals in each group from extraneous causes. Mean induction time of tumours is often used as an alternative method of comparing animal groups, but it does not distinguish between groups with different concentrations of cancer deaths along the time axis. More meaningful information can be obtained from the data by the comparison of the rates of mortality from the specific cause under consideration. This has been discussed by Pilgrim and Dowd (1963) and Murray (1965). The method described by the former authors has been used here to compare the mortality rates from breast cancer in the experimental groups of mice.

### RESULTS

*Dibenzanthracene*

The incidence and induction time of breast tumours arising in the various groups of mice after DBA treatment is given in Table I. It also shows the

TABLE I.—*Incidence and Induction Time of Breast Tumours and Survival Time of Mice maintained under different Social Conditions After Skin Paintings with Dibenz[a,h]anthracene in Olive Oil*

| | | | Breast tumours | | | | | | |
|---|---|---|---|---|---|---|---|---|---|
| | | | | | | Latent period (weeks from 1st DBA) | | DBA survival time (weeks) | |
| Genetic type | Group | Number of mice | Number with tumours | Incidence (per cent) | Number with multiple tumours | Mean | Range | Mean | Range |
| C57Bl | IV | 16 | 0 | 0 | 0 | — | — | 60 | 35–97 |
| | GV | 17 | 4 | 24 | 0 | 80 | 59–103 | 81 | 54–103 |
| | PP | 14 | 2 | 14 | 0 | 71 | 69–73 | 80 | 45–99 |
| | FB | 19 | 10 | 53 | 1 | 53 | 29–88 | 59 | 29–88 |
| | LB | 15 | 3 | 20 | 0 | 60 | 50–68 | 68 | 50–94 |
| IF | IV | 16 | 14 | 88 | 12 | 32 | 21–45 | 31 | 21–45 |
| | GV | 17 | 16 | 94 | 8 | 30 | 18–46 | 30 | 18–46 |
| | PP | 20 | 19 | 95 | 11 | 30 | 23–41 | 29 | 23–41 |
| | FB | 21 | 21 | 100 | 11 | 33 | 19–49 | 33 | 19–49 |
| | LB | 16 | 9 | 56 | 6 | 37 | 22–57 | 39 | 22–57 |
| $F_1$ (C57Bl × IF) | IV | 16 | 14 | 88 | 5 | 54 | 41–80 | 55 | 41–80 |
| | GV | 18 | 18 | 100 | 7 | 47 | 29–60 | 47 | 29–60 |
| | PP | 21 | 18 | 86 | 9 | 45 | 25–77 | 47 | 25–77 |
| | FB | 15 | 12 | 80 | 7 | 40 | 25–53 | 41 | 25–53 |
| | LB | 17 | 1 | 6 | 0 | 50 | — | 59 | 45–93 |

survival time of the various groups. High incidences of tumours were obtained in most groups of IF and hybrid mice, while generally low incidences were found in C57Bl. The latent period of tumour appearance was shortest in IF mice, somewhat longer in hybrids and very long in C57Bl.

The mortality rates from breast cancer for the 3 genetic types of mice used are shown in Fig. 1, 2 and 3.

Fig. 1 shows that the rate of tumour development in C57Bl mice was very slow after DBA treatment. Isolation of virgins resulted in no tumours developing at all. Forced breeding had quite a marked tumour-promoting effect, but lactation reduced this back to the normal level.

Fig. 2 shows that the rate of breast tumour development in IF mice after DBA was rapid. In this strain lactating breeders also showed tumour inhibition compared with forced breeders, but other social conditions had no marked effect on tumour development.

The $F_1$ hybrid (C57Bl × IF) mice showed some resemblance to both their parent strains. They had a rapid rate of tumour development (Fig. 3) similar to, though not so rapid as the IFs. Lactation was extremely inhibitory of tumour

FIG. 1.—Breast tumour mortality rate of C57Bl mice treated with dibenz[*a,h*]anthracene.

FIG. 2.—Breast tumour mortality rate of IF mice treated with dibenz[*a,h*]anthracene.

development, more so than in either parent strain. Of the other social conditions, the greatest difference was seen between the isolated virgins and the forced breeders, as in the C57Bl strain. The rate of tumour development in these 2 groups was similar (the lines run parallel) but the onset of tumours in the isolated virgins was delayed by some 14 weeks or so.

Pseudopregnant mice of none of the 3 genetic types showed any enhanced susceptibility to breast tumours after DBA treatment. This contrasts markedly with the susceptibility to tumours after MC treatment of the same types of mice.

TABLE II.—*Incidence of Pathological Lesions Found in Mice Receiving Skin Paintings of Dibenz[a,h]anthracene in Olive Oil*

| Genetic type | Total number | " Leukaemia " per cent | Liver lesions per cent | Skin tumours per cent | Ovarian tumours per cent | Lung adenomas per cent |
|---|---|---|---|---|---|---|
| C57Bl | 81 | 26 | 17 | 26 | 5 | 0 |
| IF | 90 | 6 | 3 | 4 | 0 | 2 |
| $F_1$ (C57Bl × IF) | 87 | 7 | 6 | 14 | 0 | 22 |

FIG. 3.—Breast tumour mortality rate of $F_1$ (C57Bl × IF) mice treated with dibenz[*a*,*h*]anthracene.

Table II shows the incidence of other pathological lesions most frequently found. These lesions were distributed more-or-less evenly amongst the different groups of mice within each strain, so they are reported here only by genetic type.

The " leukaemias " were nearly always lymphocytic, involving spleen, lymph nodes and often infiltrating the liver. The liver lesions referred to in the table were not associated with leukaemic infiltration. They consisted of toxic degeneration and regeneration nodules. Skin tumours had a somewhat longer latent period than breast tumours and were more slow growing. Thus they were seen particularly in the groups of mice which developed few or no breast tumours, but also occurred together with breast tumours in some animals. Ovarian tumours were small and exclusive to C57Bl mice. There were 2 granulosa-celled tumours and 2 tubular adenomas. In addition, 3 blood clots and one cystic ovary were found in C57Bl mice. Lung adenomas were seen mainly in hybrids. They occurred amongst the oldest survivors.

*Methylcholanthrene*

For comparison with the experiments reported above using DBA, the data obtained previously with similar groups of mice treated with 0·5 per cent MC in olive oil are summarised here.

Table III shows the incidence and induction time of breast tumours after MC painting, while Fig. 4, 5 and 6 show the mortality rates of the 3 genetic types of mice from breast cancer.

FIG. 4.—Breast tumour mortality rate of C57Bl mice treated with 3-methylcholanthrene.

TABLE III.—*Incidence and Induction Time of Breast Tumours and Survival Time of Mice maintained under differents Social Conditions After Skin Paintings with 3-Methylcholanthrene in Olive Oil*

| | | | Breast tumours | | | | | | |
|---|---|---|---|---|---|---|---|---|---|
| | | | | | | Latent period (weeks from 1st MC) | | MC survival time (weeks) | |
| Genetic type | Group | Number of mice | Number with tumours | Incidence (per cent) | Number with multiple tumours | Mean | Range | Mean | Range |
| C57Bl | GV | 13 | 2 | 14 | 0 | 26 | 23–29 | 38 | 23–41 |
| | PP | 21 | 11 | 52 | 0 | 36 | 24–45 | 46 | 24–83 |
| | FB | 22 | 7 | 32 | 4 | 36 | 26–50 | 39 | 26–56 |
| | LB | 18 | 7 | 39 | 2 | 41 | 34–48 | 44 | 34–57 |
| IF | IV | 15 | 14 | 93 | 12 | 25 | 19–36 | 24 | 19–36 |
| | GV | 19 | 14 | 74 | 3 | 41 | 28–82 | 46 | 28–82 |
| | PP | 20 | 19 | 95 | 12 | 19 | 15–25 | 19 | 15–25 |
| | FB | 13 | 9 | 69 | 5 | 21 | 15–28 | 23 | 15–28 |
| | LB | 18 | 0 | 0 | 0 | — | — | 39 | 19–63 |
| $F_1$ (C57Bl × IF) | IV | 28 | 21 | 75 | 9 | 35 | 26–46 | 36 | 26–56 |
| | GV | 34 | 33 | 98 | 17 | 34 | 21–52 | 34 | 21–52 |
| | PP | 32 | 30 | 94 | 20 | 24 | 18–38 | 24 | 18–38 |
| | FB | 30 | 25 | 82 | 15 | 28 | 16–43 | 29 | 16–43 |
| | LB | 32 | 3 | 9 | 1 | 30 | 25–33 | 31 | 20–41 |

High incidences of tumours were obtained with MC; the mortality rates were faster (as indicated by the steeper slopes of the lines) and the latent periods were shorter than with DBA. The different genetic types ranked similarly in sensitivity after both carcinogens.

C57Bl (Fig. 4) virgin mice were very resistant to tumour induction, but pseudopregnancy or breeding were able to increase the sensitivity of this strain. Lactation had no apparent inhibitory effect.

In IF mice, the latent periods were shorter and the rate of tumour appearance slightly faster than in hybrids (Fig. 5 and 6), but the effects of different social

FIG. 5.—Breast tumour mortality rate of IF mice treated with 3-methylcholanthrene.

FIG. 6.—Breast tumour mortality rate of $F_1$ (C57Bl × IF) mice treated with 3-methylcholanthrene.

conditions were similar in these 2 types of mice. Lactation was extremely inhibitory and pseudopregnancy had a quite marked effect in promoting the early development of tumours.

The incidence of other pathological lesions in mice painted with MC are shown in Table IV.

TABLE IV.—*Incidence of Pathological Lesions Found in Mice Receiving Skin Paintings of* 3-*Methylcholanthrene in Olive Oil*

| Genetic type | Total number | " Leukaemia " | Skin tumours per cent | Ovarian tumours per cent |
|---|---|---|---|---|
| C57Bl | 76 | 12 | 63 | 0 |
| IF | 81 | 17 | 21 | 1 |
| $F_1$ (C57Bl × IF) | 156 | 33 | 42 | 8 |

Ovarian tumours were granulosa-celled and there were 2 large ones in hybrids. There were also 9 cystic ovaries in IF mice and 1 ovarian haemangioma in both IF and C57Bl mice. Toxic degeneration of the liver was not seen with this carcinogen. Lung adenomas were not recorded. Skin tumours were more frequent than after DBA, but again they were more slow growing than breast tumours so tended to occur in the longest survivors of groups which developed few or no breast tumours.

### DISCUSSION

The results of the present experiments confirm the previous findings of Bonser (1958), Jull (1958 and 1964), Biancifiori, Bonser and Caschera (1961) and Ranadive and Karande (1963) that dibenz[*a*,*h*]anthracene is a powerful breast carcinogen for mice. Ranadive and Karande, using 0·25 per cent solutions in benezene biweekly, considered DBA to be more powerful than MC. However, the present experiments, using 0·5 per cent in olive oil, indicate that DBA produces breast tumours at a slightly slower rate (Fig. 1–6) and with a longer latent period than MC in similar types of mice (Tables I and III). They confirm Bonser's earlier findings in IF virgins. Biancifiori *et al.* (1961) also obtained fewer breast tumours in C3Hb mice after administration of DBA solutions by stomach tube than after administration of MC in similar doses.

It is evident that the genetic constitution of the mice is of the greatest importance in determining the sensitivity to breast cancer induction by DBA. Each type used in the present experiments responded in its own particular way, developing breast tumours at a rate which was characteristic of the strain. Although the latent period of tumour appearance might be notably changed, the rate was relatively little affected by different social conditions, except in a few extreme cases. However, the characteristic rate of breast tumour appearance for each strain does not appear to be determined by genetic factors acting at the level of the breast tissue itself, for Riggott (1965) has shown that breasts from IF, C57Bl or $F_1$ (C57Bl × IF) donors transplanted to $F_1$ (C57Bl × IF) hosts all respond to tumour induction by MC in a manner characteristic of the host mice. Evidently the response is mediated through the internal environment of the mouse.

Ranadive and Karande (1963) studied the response of 5 different strains of mice to breast tumour induction by DBA. Two of these were complicted by the presence of MTA, but the other 3 strains varied greatly in sensitivity. DBA

virgins were very sensitive, while C57Bl and L(P) virgins were resistant. Breeding did not change the sensitivity of C57Bl, but L(P) breeders were very sensitive. Jull (1964) has studied the effect of several different social conditions on breast tumour induction by DBA in C3Hb mice. He found that virgins developed breast tumours in small numbers after lengthy latent periods. The administration of oestrone to virgins increased their sensitivity, but pseudopregnancy had a more marked enhancing effect. Forced breeding was less effective and lactation had no particular inhibitory effect in these mice. C3Hb mice, then, behave very differently from the mice used in the present experiments, which all showed tumour inhibition by lactation and no enhancement by pseudopregnancy.

At present the situation is confusing. It is impossible to make any definite statements about the effect of particular social conditions on susceptibility to breast tumour induction by this or that carcinogen. Each genetic type responds in its own way to a particular carcinogen and may respond in a somewhat different way to another carcinogen. Different social conditions with their accompanying hormonal disturbances do not always have the same kind of effects on breast tumour induction in different genetic types of mice. Clearly, much more detailed comparisons need to be made between mammary carcinogenesis by MC and DBA to establish whether, or not, Jull's suggestion that they work in fundamentally different ways can be substantiated. It is interesting that DBA appears to induce breast tumours with less tendency to squamous metaplasia than MC. Also that DBA seems to have the most marked effect on ovaries of C57Bl mice, while MC affects ovaries of mice with IF parentage.

### SUMMARY

Mice of 3 genetic types, maintained under different social conditions, were given 8 skin paintings of 0·5 ml. saturated solution of dibenz[*a,h*]anthracene in olive oil at fortnightly intervals.

Breast tumours appeared with rates characteristic for each genetic type. In C57Bl they were slow to appear, with a long latent period, but were enhanced by forced breeding. In IF mice they appeared at the most rapid rate, with the shortest latent period, and in this strain lactation was inhibitory. In the $F_1$ (C57Bl × IF) hybrids the rate of appearance was slower than in IF mice, the latent period was longer and lactation was extremely inhibitory.

The results are compared with those previously obtained after treatment of similar mice with 3-methylcholanthrene.

I wish to thank the Birmingham Branch of the British Empire Cancer Campaign for Research for support of this work.

### REFERENCES

Biancifiori, C., Bonser, G. M. and Caschera, F.—(1961) *Br. J. Cancer*, **15**, 270.

Bonser, G. M.—(1954) *J. Path. Bact.*, **68**, 531.—(1958) in ‘ International Symposium on Mammary Cancer ’ edited by L. Severi, Perugia, p. 575.

Howell, J. S., Marchant, J. and Orr, J. W.—(1954) *Br. J. Cancer*, **8**, 635.

Jull, J. W.—(1954) *J. Path. Bact.*, **68**, 547.—(1956) *Acta Un. int. Cancr.*, **12**, 623.—(1958) in ‘ International Symposium on Mammary Cancer ’, edited by L. Severi, Perugia, p. 423.—(1964) *Br. J. Cancer*, **18**, 508.

MARCHANT, J.—(1963) *Br. J. Cancer*, **17**, 495.—(1964) *Acta Un. int. Cancr*, **20**, 1443.
MÜHLBOCK, O. AND BOOT, L. M.—(1961) *Natn. Cancer Inst. Monog.*, **4**, ' Symposium on phenomena of the tumour viruses ', p. 129.
MURRAY, W. S.—(1965) *J. natn. Cancer Inst.*, **34**, 21.
ORR, J. W.—(1943) *J. Path. Bact.*, **55**, 483.
PERRY, I. H. AND GINZTON, L. L.—(1937) *Am. J. Cancer*, **29**, 680.
PILGRIM, H. I. AND DOWD, J. E.—(1963) *Cancer Res.*, **23**, 45.
RANADIVE, K. J. AND KARANDE, K. A.—(1963) *Br. J. Cancer*, **17**, 272.
RIGGOTT, J. M.—(1965) *Br. J. Cancer*, **19**, 174.
SYDNOR, K. L. AND COCKRELL, B.—(1963) *Endocrinology*, **73**, 427.
VAN DER LEE, S. AND BOOT, L. M.—(1955) *Acta physiol. pharmac. néerl.*, **4**, 442.